# RESTART: a stepped-care approach to facilitate return to work for employees with psychological distress: design of a randomized controlled trial

**DOI:** 10.1186/s12889-024-19773-3

**Published:** 2024-08-22

**Authors:** Hanneke A.M. Lettinga, Sandra H. van Oostrom, Hendrika P. Zijlstra, Johannes R. Anema, Karin I. Proper

**Affiliations:** 1 Center for Prevention, Lifestyle and Health, National Institute for Public Health and the Environment, Bilthoven, The Netherlands; 2 Department of Public and Occupational Health, Amsterdam UMC, Vrije Universiteit Amsterdam, Amsterdam Public Health Research Institute, Amsterdam, The Netherlands; 3 Zorg van de Zaak Netwerk, Utrecht, The Netherlands

**Keywords:** e-Health, Psychological distress, Participatory approach, Return to work, Stepped-care, Workplace intervention, Mental health

## Abstract

**Background:**

Common mental health problems, such as stress, anxiety and depression, are highly prevalent among workers and often lead to long-term absenteeism and work disability. Effective elements found in previous researched interventions were to explicitly focus on return to work (RTW) and not solely on symptom reduction, to take into account the employees’ cognition towards RTW and to include the workplace environment. Based on these elements, a stepped-care approach was developed. The aim of this paper is to present the study design of a randomized controlled trial (RESTART), evaluating the effectiveness of the stepped-care approach on lasting RTW and the implementation process.

**Methods:**

RESTART is a randomized controlled trial with a 2 × 2 factorial design and a follow-up of one year. Employees eligible for this study are those who reported sick within 2 to 8 weeks with psychological distress based on a distress screener. Participants will be randomized to a group receiving a tailored e-Health app or usual care, as well as randomized to a group receiving a Participatory Approach (PA; conversational method) in the workplace or usual care. The PA will however only be provided in case of persistent sickness absence at 8 weeks. Measurements take place at baseline, after the e-Health intervention period (3 months), and after the PA intervention period (6 months) and 12 months. Primary outcome is lasting RTW, defined as full RTW in previous or equal work for at least four consecutive weeks. Secondary outcomes are (the severity of) stress-related symptoms, total number of sickness absence days, self-efficacy for RTW and self-reported health. A process evaluation including a realist evaluation will also be conducted.

**Discussion:**

Early intervention that focuses on RTW, the cognition towards RTW despite symptoms and involves the workplace environment, plays a crucial role in managing sickness absence among employees with psychological distress. If effective, the stepped-care approach is relevant for employees, employers and society as a whole.

**Trial Registration:**

ISRCTN: 90663076. Registered on 5 October 2023.

**Supplementary Information:**

The online version contains supplementary material available at 10.1186/s12889-024-19773-3.

## Background

Common mental health problems, such as depressed mood, anxiety and stress, are of growing concern in today’s workforce. Common mental health problems affect the well-being, productivity and economic prosperity of individuals, organizations and society as a whole [1]. In 2019, 42% of sickness absence days in the Netherlands were caused by common mental health problems [2]. As work provides structure in one’s day, social contacts and contributes to a feeling of appreciation, not being able to work due to common mental health problems has a huge impact on the employee, together with being at high risk for long-term absenteeism [3, 4]. The employer and society are confronted with high costs due to absenteeism and incapacity to work caused by common mental health problems. Recently, a Dutch study showed that on average, one episode of sickness absence due to stress-related health problems lasted 101 working days, which led to costs for the employer of above 19 thousand euros [3]. It is also known that the probability of eventual return to work (RTW) declines with longer absence [5], which poses a challenge for absenteeism due to common mental health problems because of its long-term nature.

When employees on sickness absence with common mental health problems currently receive treatment, these treatments mainly focus on reducing symptoms [6]. However, research has shown that a decrease of symptoms does not imply an immediate (partial) RTW [6–8]. Consequently, there has been a growing recognition of the importance of promoting RTW as a key goal of treatment and involve the workplace environment in the rehabilitation of employees with common mental health problems [9–12]. This is also driven by the emerging importance of offering interventions that provide the greatest patient value [13] and being able to work is considered one of those values [8, 14].

Some previously evaluated interventions that aimed to facilitate RTW for employees with common mental health problems showed mixed results. An e-Health program to facilitate RTW based on psychological principles, such as psycho-education, cognitive behavioral therapy (CBT) and problem-solving therapy (PST) was effective on duration to first RTW (50 days) compared to conventional sickness guidance (usual care; 77 days). The e-Health program was not effective on lasting RTW [11]. A process evaluation of the e-Health program revealed that adherence of employees to the program varied between 13 and 90% [15], which might have impacted the full realization of the programs potential.

Another study evaluated the effectiveness of a Participatory Approach (PA) for employees on sickness absence with common mental disorders [12]. The PA is a stepwise process between the employee and his or her supervisor with the aim to reach consensus about obstacles and solutions to enable RTW, under guidance of a RTW-coordinator. Overall, the PA had no effect on lasting RTW, except for the subgroup of employees who indicated at baseline that they were open to RTW despite symptoms. For these employees, with a positive cognition towards RTW despite symptoms, the PA significantly reduced the time to lasting RTW (55 days) compared to conventional sickness guidance (usual care; 120 days) [12].

As the e-Health program was effective on first RTW – but not lasting RTW, and the PA was effective only for the subgroup who had a positive cognition towards RTW despite symptoms, we propose a stepped-care approach to combine these effective elements. The stepped-care approach starts with a low-intensive e-Health early in the absenteeism process. The e-Health consists of a tailored program which focuses on developing and reinforcing a positive cognition towards RTW. If RTW is not reached, the e-Health program is followed by a more intensive PA that has greater involvement of the workplace environment. The combination of these elements in a stepped-care approach aiming to facilitate a timely and lasting RTW is considered potentially effective. The aim of this paper is to describe the design of the RESTART (*R*eturn to work for *E*mployees with distress: a *ST*epped c*AR*e *T*reatment) study, which includes an evaluation of the effectiveness of the stepped-care intervention on lasting RTW by a randomized controlled trial and a process evaluation.

## Methods

### Design and setting

The study is a randomized controlled trial (RCT) with a 2 × 2 factorial design. This design allows to test the main effects of the e-Health and PA separately, as well as their combined effect on lasting RTW. The study includes a baseline assessment and 3 follow-up assessments at 3 months (after e-Health), 6 months (after PA) and 12 months. Afterwards a process evaluation will be conducted following the Steckler and Linnan framework [16, 17], including a realist evaluation [18]. The process evaluation aims to provide insights in what elements of the stepped-care approach work, for whom and under which circumstances and to evaluate to what degree the implementation of the stepped-care approach was conducted as intended.

The study will be carried out in a large Dutch occupational health service (OHS) organization. The OHS consists of about 4,000 employees – mainly white-collar workers. Occupational health (OH) practitioners from the OHS organization, which has multiple branches spread over the Netherlands, are involved in this study. The core business of the OHS is to offer services to different organizations with the goal to increase the health of workers, promote sustainable employability, prevent sick leave, and offer support for RTW after sickness absence. The task of the OP in the Netherlands is to prevent work-related diseases and to support workability and RTW after sickness absence. The OH practitioner collaborates with the OP and works in task delegation of the OP to reduce the work pressure of the OP. Both OPs and OH practitioners have consultations with employees to promote workplace health, to prevent sickness absence, or to support RTW. Participants will be recruited within the OHS organization itself and their partners.

The trial has been approved by the Medical Ethics Committee of the Amsterdam Academic Medical Center (2023.0474) and is registered in the ISRCTN registry (ISRCTN90663076). Signed informed consents will be obtained from each participant.

### Study population

Participants will be employees of the OHS network, who reported sick for a minimum of 2 weeks and a maximum of 8 weeks. Participants are eligible if they (1) filled in the distress screener based on the Four-Dimensional Symptoms Questionnaire (4DSQ), (2) met the distress criteria and (3) signed informed consent.

Exclusion criteria will be (1) severe psychiatric disorders (suicidal risk, schizophrenia, bipolar disorder), (2) treatment for terminal or chronic illnesses that preclude their ability to resume work in the near future (e.g. chemotherapy or cardiac surgery), (3) a labour dispute between the employee and the employer, involving legal action, (4) working less than 12 h per week according to contract, (5) pregnancy, (6) no proficiency of the Dutch language and (7) no access to the internet.

Within the literature, different terminology and definitions are used for describing (different subgroups of) mental health problems. For example, common mental disorders, mental health problems, psychological complaints and distress are used. In the current study we will refer to employees with psychological distress. Psychological distress is defined as self-reported complaints of non-specific stress symptoms, depressed mood, anxiety and/or somatization.

### Recruitment and procedures

Recruitment will start in April 2024, and will be ongoing until the necessary number of participants is reached. Within one week of absenteeism, the employee will be notified of the study by means of a digital information letter that is automatically sent to the employee via the absence registration process of the OH physician. The digital information letter will contain all required information about the study and a QR-code (and website link) that will lead to a safe online questionnaire that contains the distress screener and questions regarding the exclusion criteria. The distress screener, derived from the 4DSQ [19], consists of three questions and is a valid instrument for identification of distress in employees on sickness absence [20]. If the employee is eligible and willing to participate, they will receive the informed consent form digitally from the researchers, together with the possibility to schedule a (video)call with the researcher or the OH practitioners in case they require more information before they want to participate, i.e. sign informed consent. After online informed consent is obtained, the participant is invited to the online baseline assessment.

### Randomization and blinding

**Fig. 1:**
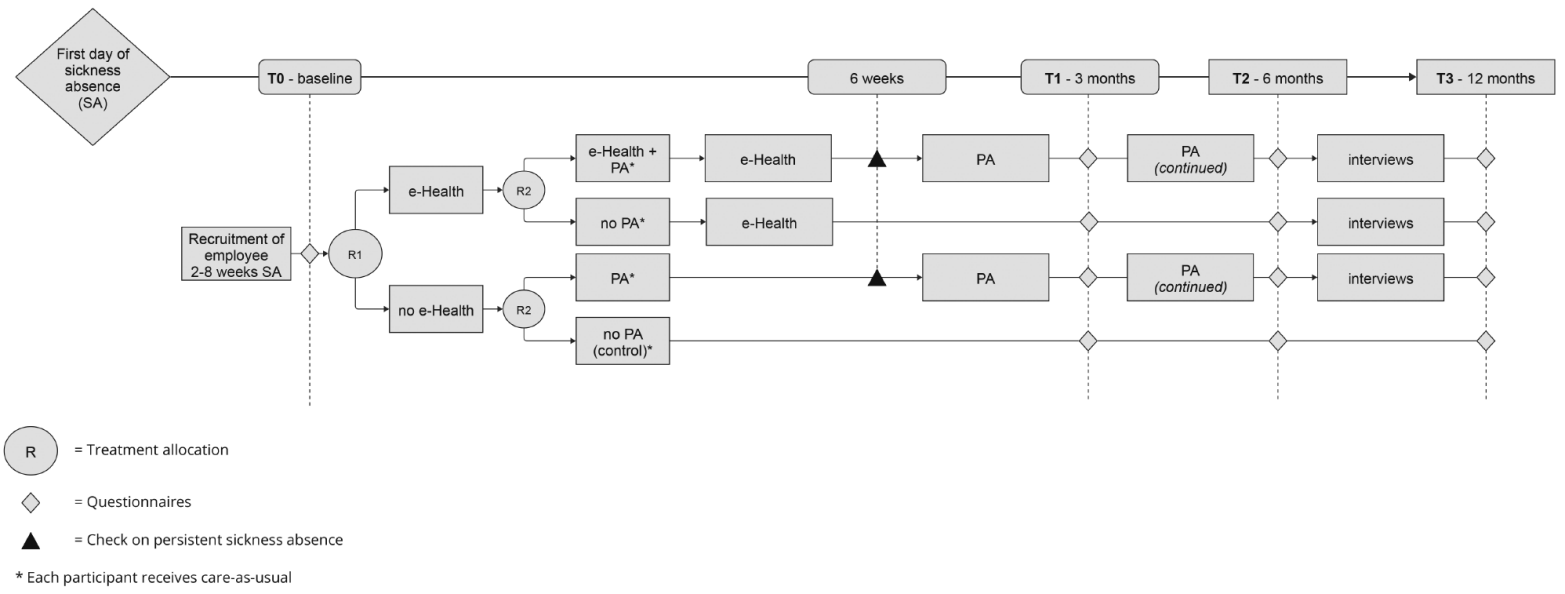
Time schedule of the RCT

**Fig. 2:**
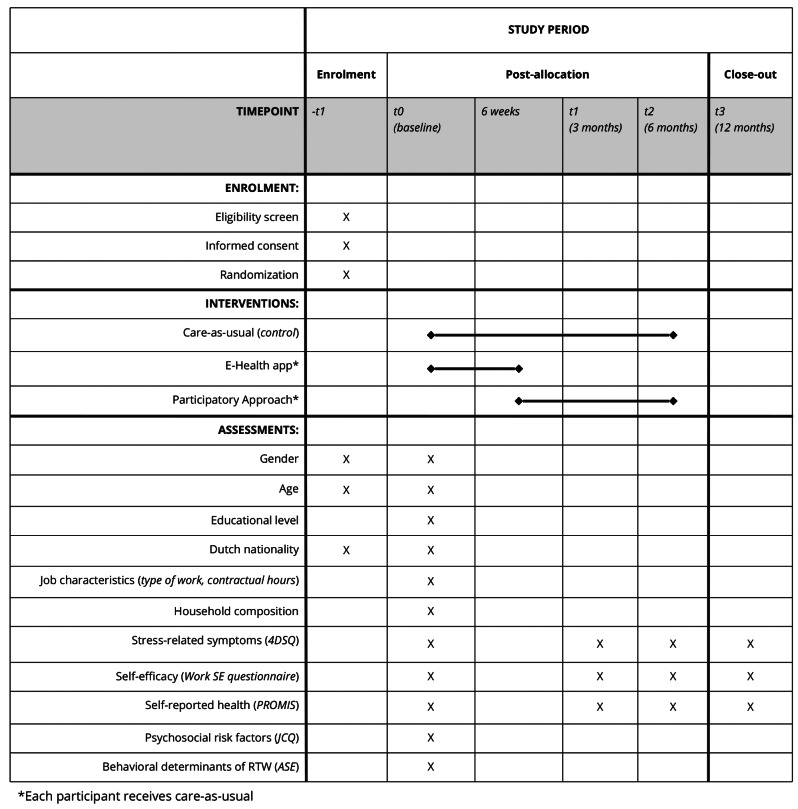
SPIRIT diagram depicting participant timeline

Randomization takes place on employee level. In addition to usual care, participants can receive e-Health and/or PA. This leads to a 2 × 2 factorial design, which allows to test the main effects of e-Health and PA separately, as well as the combined effect on lasting RTW. Therefore, participants are randomized twice, which results in two comparisons in the main outcome analysis: PA vs. not PA and e-Health vs. no e-Health. On top of usual care participants receive either: (1) e-Health and PA, (2) e-Health only, or (3) PA only (see Fig. 1). A time schedule of enrolment, interventions, and assessments are presented in Fig. 2. Both randomizations take place directly after baseline to ensure even-sized groups. If, however, participants who are assigned to receiving PA (partially) returned to work before PA starts, they will not receive PA.

A computer-based randomization list, generated by an independent researcher, will be used to allocate participants, based on their participant-ID, to one of the four groups. Before the statistical analyses are performed the condition-IDs are recoded by an independent researcher, which allows the primary researcher to be blinded for the allocation and the RCT to be single-blind.

### Sample size

The sample size was calculated based on number of cases needed to identify an effect on time to (lasting) RTW. Based on previous research of RTW interventions for employees with psychological distress a hazard ratio (HR) was chosen of 1.8 [11, 12, 21]. A sample size calculation was performed with an HR of 1.8, alpha of 0.5 and power of 80%, which generated a total sample size of 144 participants, 36 participants per group. Accounting for a loss-to-follow up of 15% after 12 months, we aim to include 166 participants.

### Interventions

All participants receive usual care which consists of conventional sickness guidance by the OP, following the guidelines of the Netherlands Society of Occupational Medicine (NVAB). All guidance within the interventions (e-Health and PA) are performed by trained RTW-coordinators. Three OH practitioners received a one day training by the researchers in this role.

#### e-Health intervention

The e-Health intervention is based on the original Return@Work intervention [11], and adjusted according to the guidelines for common mental disorders of the Netherlands Society of Occupational Medicine [22]. After completing the baseline assessment, employees allocated to the e-Health group will receive access to the e-Health app. The employee is provided with a personal log-in code for the app. The e-Health application starts with an introduction on how to use the app and asks for the necessary permissions of the employee. Following this, the employee answers two navigating questions. The first navigating question is whether or not the employee believes he/she can return to work despite symptoms (cognition of RTW). If this question is followed by a negative answer (i.e. maybe/probably not/no/don’t know), this will be taken as an indication of a negative cognition towards RTW – and the program will include extra exercises to create and reinforce a positive cognition towards RTW. The second navigating question is if the employee labels his/her complaints as more “psychological” or more “physical”. In certain cases, employees with psychological distress categorize their complaints as primarily physical rather than psychological. In such cases, using psychological terminology usually doesn’t resonate with that employee’s experience which can lead to a lower adherence to the program. Therefore, if the employee labels their complaints as more physical, the information that is provided in each module will also be in terms of physical complaints.

Based on the scores of the 4DSQ and the navigating questions, the employee receives specific modules tailored to their needs.

The e-Health app includes the following modules:

- Psycho-education on the relationship between psychological distress and RTW.
- Exercises aimed at creating and/or reinforcing a positive cognition towards RTW despite symptoms, based on cognitive behavioral principles.
- Psycho-education on pain and fatigue and the relationship with psychological distress, work and RTW. Exercises are based on relaxation principles.
- A task-analysis of all activities the employee performs in his/her work, and in which of these activities they foresee/experience obstacles for RTW.
- Problem-solving exercises specifically for the process of RTW. The steps include defining problems and goals, learning and applying problem-solving skills and developing more control of a problem-situation.
- Preventing relapse, aiming to make the employee more aware of personal distress signals that could increase the likelihood of loss of control.

The total duration of the e-Health is 6 weeks. Based on the findings of the process evaluation of Return@Work [15] and previous research about engagement in e-Health [23], employees will be stimulated to engage with the e-Health application to increase the likelihood that the employee will complete the program within 6 weeks. These means include timely notifications and reminders, personal progression of the modules and “achievements” after finishing modules, and the possibility to have a 30 min consult with one of the OH practitioners during the e-Health program.

#### Participatory approach (PA)

The Participatory approach (PA) is coordinated by one of the independent RTW-coordinators. Participants will receive PA six weeks after baseline; after completing the e-Health program or after care-as-usual, depending on their respective condition. The RTW-coordinator plans three meetings: the first meeting with the employee, the second meeting with his or her supervisor and the third meeting with the employee and supervisor. These meetings are scheduled shortly after each other (e.g. within two weeks).

In the first meeting, the employee is guided by the RTW-coordinator to perform a task-analysis and obstacle inventory. In a guided conversation, the employee first lists their main responsibilities and activities in their work, and uses this list to identify obstacles for RTW. The first meeting finishes after ranking the obstacles for RTW based on priority, frequency and perceived severity. If the employee received e-Health they already practiced with this and they can use that list to refine obstacles together with the RTW-coordinator. At the second meeting, the task-analysis and obstacle inventory are performed from the perspective of the supervisor, together with the RTW-coordinator. In the third meeting, the employee and his or her supervisor discuss the identified obstacles and brainstorm about solutions for these obstacles. Under guidance of the RTW-coordinator, they will rank the solutions, based on feasibility, solving capability and short-term applicability. The third meeting concludes with a RTW plan. This RTW plan includes the chosen solutions based on consensus between the employee and the supervisor, and is formulated in terms of who is responsible, to do what and when. At the end of the meeting, the RTW-coordinator plans an evaluation moment with the employee and the supervisor. The chosen solutions are to be implemented in the weeks after the third meeting, ideally within a time frame of 3 months. After three months the actual implementation of the solutions will be evaluated by the RTW-coordinator, together with the employee and the supervisor.

### Outcome measures

#### Effect evaluation

The primary outcome measure is time to lasting RTW. Lasting RTW is defined as RTW to the employee’s previous position, or another position with equal earnings, for a minimum of four consecutive weeks. Time between the first day of absenteeism with psychological distress and lasting RTW is calculated in calendar days and will be based on self-report through monthly diaries that participants are asked to fill in online. In addition, permission was requested to obtain these data from the OHS.

The secondary outcome measures consist of (1) (severity of) stress-related symptoms, (2) total sick-leave days, (3) self-efficacy and (4) self-reported health. Stress-related symptoms are measured with the 4DSQ [19, 24]. This questionnaire consists of 50 items, with subscales distress, depression, anxiety, and somatization. The items are scored on occurrence in the past week on a 5-point Likert scale, ranging from “no complaints” to “very often/continuously”. The total number of sick-leave days is based on the same data as time to lasting RTW (self-report and/or absenteeism record). Self-efficacy with regard to RTW is measured using the Work Self-Efficacy questionnaire. This questionnaire consists of 11 items and is specifically designed to measure self-efficacy for RTW in employees with mental health problems [25]. Self-reported health is measured with the Patient Reported Outcomes Measurement Information System (PROMIS) questionnaire. The PROMIS covers three domains of reported health: physical, psychological and social aspects of wellbeing. [26].

#### Covariates

By means of questionnaires we will gather information on the following covariates: (1) sociodemographic data, (2) job characteristics, (3) psychosocial risk-factors, (4) behavioral determinants of RTW. Sociodemographic data will include age, sex as reported by the employee, educational level and household composition. Job characteristics include type of work and contractual hours. Psychosocial risk-factors will be measured using the Job Content Questionnaire (JCQ; [27]. This questionnaire is designed to measure the social and psychological characteristics of jobs, including psychological demands, decision latitude (the employees’ potential control over his tasks and conduct during the workday), social support, physical demands and job insecurity. Behavioral determinants of RTW following the ASE model consist of the employees’ attitude, social influence and self-efficacy [28, 29].

#### Data analysis

Descriptive statistics will be provided for all relevant variables at baseline. To investigate the effectiveness of the interventions of the stepped care approach on lasting RTW a cox-regression will be conducted. To determine the effect of e-Health separately, the group who received [e-Health and PA] will be combined with the [e-Health only] group, and compared to the control- and [PA only] group. The same logic applies to PA; the PA groups will be combined and compared to the control- and [e-Health only] group. To test whether the combination of e-Health and PA in a stepped-care approach is superior to the separate interventions alone, we will study the interaction between PA and e-Health. For the secondary outcomes, depending on the type of outcome measure (continuous/categorical) generalized linear models or mixed effects logistic regression will be used. Analyses are conducted both unadjusted and adjusted for potential confounders. Additionally, effect modification of several factors, such as the (severity of) stress-related symptoms and cognition towards RTW despite symptoms, will be investigated by using interaction terms.

All analyses will be performed according to the intention-to-treat principle (ITT), which implies that all employees are analyzed according to the group they were assigned to, regardless of whether they actually received the intervention.

#### Process evaluation

A process evaluation will be conducted based on the Steckler and Linnan framework [16, 17], including a realist evaluation [18]. The goal of the process evaluation is to identify underlying mechanisms that lead to success or failure of (parts of) the stepped-care approach. Depending on the effectiveness of the stepped-care approach, it will be used to answer questions about “what works, for whom, in what contexts in what respects and how” [30]. The process evaluation consists of two components: a realist evaluation and the evaluation that follows the guide of Saunders et al. [17] – which is based on the Steckler and Linnan framework. Typically, a realist evaluation is expressed in the form of context-mechanism-outcome (CMO) configurations. These configurations will be formed by gathering information about the experience with the stepped-care approach using mixed methods, after the intervention took place [31]. For example, a participant who received the stepped-care approach will be interviewed on what elements did/didn’t work – and why they think that was the case. This information is then used to form CMO’s for that participant.

For the second part of the process evaluation we will follow the guide by Saunders et al. [17] to investigate the implementation of the stepped-care approach. This evaluation will consist of six key components: fidelity, dose delivered, dose received in terms of exposure, dose received in terms of satisfaction, reach, recruitment and context.

- *Fidelity* refers to the extent to which the stepped-care approach was implemented according to the pre-specified plan.
- *Dose delivered* relates to the degree which employees who were supposed to receive the stepped-care approach (or one of its elements) actually received those.
- *Dose received in terms of exposure* refers to the extent to which employees actively engaged in the (the different components of the) stepped-care approach.
- *Dose received in terms of satisfaction* refer to the satisfaction with the stepped-care approach of all stakeholders (employees, employer and OH practitioner).
- *Reach* considers the degree to which employees on sickness absence with psychological distress participate in the intervention.
- *Recruitment* concerns the procedures of how the employees are recruited, and the reasons for not participating in the study.
- *Context* refers to the environmental or organizational factors that may impede or facilitate the intervention implementation.

For both components of the process evaluation, data will be gathered using mixed methods. At approximately 6 months (T2), when the stepped-care approach is finished, employees in one of the three experimental conditions (e-Health and PA, e-Health only, PA only) will receive additional questions to measure their experience with the stepped-care approach and the separate elements. In addition, semi-structured interviews will be held separately with 8 to 10 employees, 5 supervisors, the two RTW-coordinators and 3 to 5 OPs. The interviews will be audio-recorded and transcribed verbatim, coding and analyses will be performed using MAXQDA software. Further, quantitative information from the app including app usage data, attendance logs will be collected and analyzed, as well as the information from the forms concerning the PA.

## Discussion

In the RESTART study, we will evaluate the effectiveness of a stepped-care approach on lasting RTW for employees on sickness absence with psychological distress. We will examine the main effects as well as the combined effect of the e-Health and PA on time to lasting RTW. We hypothesize that the stepped-care approach, e-Health followed by PA, will be more effective on time to lasting RTW compared with usual care, and e-Health or PA only.

As has been shown in previous research, there are three identified key elements for successful lasting RTW for employees with psychological distress. These include an explicit focus on RTW and not only on reduction of symptoms, having a positive cognition towards RTW and involvement of the workplace environment [11, 12]. In the RESTART study, we combine these three elements in a new stepped-care approach. The first step – the e-Health program – aims to develop and reinforce a positive cognition towards RTW through different modules tailored to the employee’s needs. With persistent sickness absence the program is followed by the second step – the PA – involving the workplace environment in a more substantial manner. Since the PA has shown to be more effective for employees with a positive cognition towards RTW and the aim of the e-Health program is to develop and reinforce such a positive cognition, the two interventions in the stepped-care approach complement each other.

### Strengths and limitation

The 2 × 2 factorial design allows to test the main effects of the e-Health and PA separately, and the combined effect on lasting RTW, while keeping the sample size relatively small. A possible limitation of the 2 × 2 factorial design is that it requires both randomizations directly after baseline, but it is unknown how many employees eventually will complete PA, as this depends on persistent sickness absence of at least 6 weeks after baseline.

Another potential limitation of the current study could be non-adherence to the e-Health program. The e-Health program is mainly completed independently by the employee, and therefore relies more heavily on individual motivation. To minimize the chance of non-adherence employees will be stimulated to engage with the program [23] and are offered the possibility to have a 30 min consult with the OH practitioner. Furthermore, the patterns of app usage and adherence to the program will be investigated by means of the process evaluation.

The realist evaluation and the process evaluation will provide a deeper understanding of what elements are a success or failure, and why. This will provide important knowledge for further development and implementation of the stepped-care approach in practice.

The results from the effect- and process evaluations, together with perspectives from all stakeholders (employees, employers and OH practitioners), will provide a broad insight in the effectiveness of the stepped-care approach and its elements on lasting RTW for employees on sickness absence with psychological distress. These insights can be of large potential value to employees, employers and occupational practice.

### Electronic supplementary material

Below is the link to the electronic supplementary material.

Supplementary Material 1

## Data Availability

Data sharing is not applicable to this article as no datasets were generated or analyzed during the current study.

## Abbreviations

RTW: Return to work
PA: Participatory approach
CBT: Cognitive behavioral therapy
PST: Problem-solving therapy
RCT: Randomized controlled trial
OHS: Occupational health service
OP: Occupational physician
OH: Occupational health
4DSQ: Four-dimensional symptoms questionnaire
HR: Hazard ratio
NVAB: Netherlands society of occupational medicine
PROMIS: Patient reported outcomes measurement information system
JCQ: Job content questionnaire
ASE: Attitude, social influence and self-efficacy
ITT: Intention-to-treat
CMO: Context-mechanism-outcome
